# Passive water ascent in a tall, scalable synthetic tree

**DOI:** 10.1038/s41598-019-57109-z

**Published:** 2020-01-14

**Authors:** Weiwei Shi, Richard M. Dalrymple, Collin J. McKenny, David S. Morrow, Ziad T. Rashed, Daniel A. Surinach, Jonathan B. Boreyko

**Affiliations:** 1 Department of Biomedical Engineering and Mechanics, Virginia Tech, Blacksburg, Virginia 24061 United States; 2 Department of Mechanical Engineering, Virginia Tech, Blacksburg, Virginia 24061 United States

**Keywords:** Mechanical engineering, Applied physics, Fluid dynamics, Nanoscale materials

## Abstract

The transpiration cycle in trees is powered by a negative water potential generated within the leaves, which pumps water up a dense array of xylem conduits. Synthetic trees can mimic this transpiration cycle, but have been confined to pumping water across a single microcapillary or microfluidic channels. Here, we fabricated tall synthetic trees where water ascends up an array of large diameter conduits, to enable transpiration at the same macroscopic scale as natural trees. An array of 19 tubes of millimetric diameter were embedded inside of a nanoporous ceramic disk on one end, while their free end was submerged in a water reservoir. After saturating the synthetic tree by boiling it underwater, water can flow continuously up the tubes even when the ceramic disk was elevated over 3 m above the reservoir. A theory is developed to reveal two distinct modes of transpiration: an evaporation-limited regime and a flow-limited regime.

## Introduction

When water evaporates from the nanoporous leaves of plants, the water potential within the leaves becomes negative which pulls water up the xylem conduits to sustain hydration^1–7^. According to the cohesion-tension theory^8–16^, even an absolute negative water pressure can be maintained in a thermodynamically metastable state and transmitted along continuous water columns to the root apices. This negative pressure is generated to balance the mismatch in chemical potential between the subsaturated air and saturated water within the leaf^3^.

**Figure 1:**
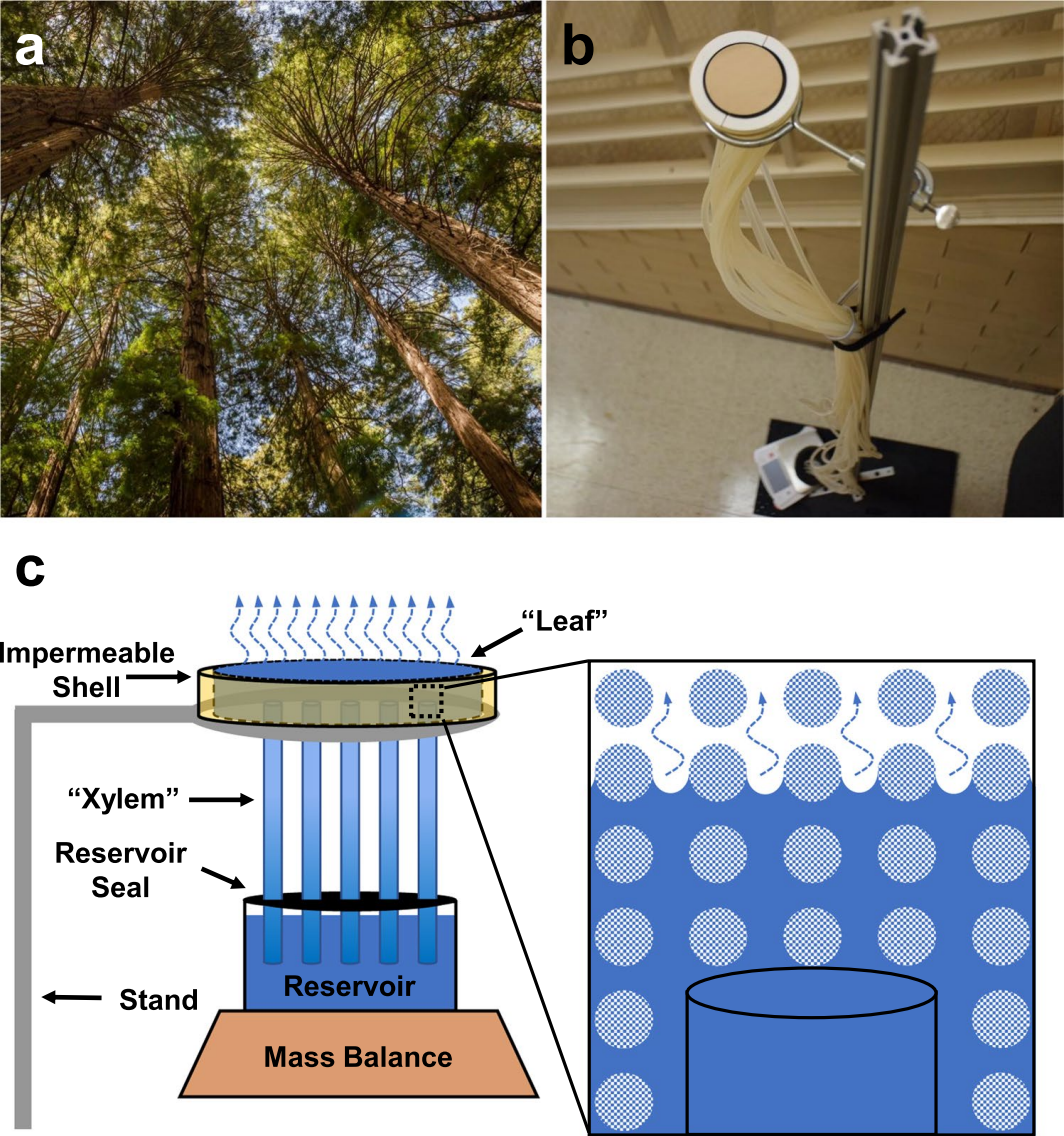
Working principles of transpiration in natural and synthetic trees. (**a**) Redwood trees, photographed at the Muir Woods National Monument in California, are exemplars of using transpiration to pump water against gravity. (**b**) Photograph of our synthetic tree setup, where water is similarly pumped against gravity and up multiple conduits in parallel. (**c**) Schematic of our scalable synthetic tree design. A negative gauge pressure is generated by the concave curvature of menisci evaporating within a nanoporous “leaf” (magnified inset), which pumps degassed water from a reservoir up a parallel array of tubes to replenish hydration.

For over a century, scientists inspired by nature’s transpiration cycle have built synthetic trees that sustain a column of liquid water^17–20^. Unlike natural trees, where the negative leaf pressure is used to pump water up a parallel network of xylem conduits, to date synthetic trees have only used a single microcapillary tube. In 1895, Dixon and Joly attained transpiration in a 0.9 m tall glass tube attached to porous cups on both ends^17^, but the single tube was only 150 *μ*m in diameter. In 1928, Thut evaporated water from a porous cylinder attached to a 1 mm-diameter glass tube terminating in a mercury reservoir; the resulting water tension elevated the mercury 2.3 m within the tube^18^. Hayward demonstrated that the liquid phase of a column of water rising 17 m above an atmospheric reservoir is stable, despite its negative absolute pressure, but this column was sustained by an active suction pump rather than by transpiration^19^. More recently, Lamb *et al*. demonstrated an 8 m column of water in a 500 *μ*m-diameter microcapillary tube connected to a microporous ceramic disk^21^. Finally, Lee *et al*. showed that the transpiration rate up a 346 *μ*m diameter tube connected to cryo-agarose gel can be slowed by replacing a pure water reservoir with high viscosity silicone oils^20^. While the heights of these one-dimensional trees are impressive, their common design of a single microcapillary severely constrains the water throughput rate.

In addition to vertically-oriented, one-dimensional synthetic trees, recent works have exploited advances in photolithographic techniques to fabricate “tree-on-a-chip” devices. Pioneering tree-on-a-chip devices continuously pumped water across pre-filled microfluidic channels using the suction power generated by the evaporation of the terminal menisci^22,23^ or by connecting filled channels to micropores exposed to subsaturated air^24^. The suction pressures generated by these micro-scale menisci were quite modest; in 2008, Wheeler and Stroock instead evaporated water from a hydrogel containing nanoscale pores to achieve hydraulic loads of Δ*P* ~ −1 MPa across an embedded microchannel^25^. In the same year, Noblin *et al*. evaporated water from PDMS to pump water across an array of microfluidic channels^26^. Several follow-up papers characterized the thermophysical properties of the negative pressure water, including its metastability^27^, cavitation dynamics^28–31^, and using nanoporous silicon (≈3 nm diameter pores) to achieve up to 100 MPa of negative Laplace pressure^32–34^. For all of these tree-on-a-chip devices, transpiration is no longer used to pump water against gravity, but rather to drive flows in micro-channels or as a platform for studying the fundamental properties of negative pressure water. Recently, a tree-on-a-chip device was shown to achieve sugar transport in synthetic phloem^35^, complementing the usual focus on water transpiration.

In summary, to date synthetic trees have either pumped water against gravity but were constrained to a single microcapillary, or were confined within substrate-bound microfluidic chips. In either case, this renders them incapable of elevating water at the high mass flow rates achieved by large, natural trees (Fig. 1a). Here, we develop a synthetic tree that is both tall and scalable to any cross-sectional area (Fig. 1b), by coupling a nanoporous ceramic disk to a parallel array of long (≈3 m) and high-conductance (≈3 mm-diameter) tubes. The water assumes a negative (gauge) pressure as it evaporates from the nanoporous disk, such that hydration is maintained by lifting water up the tube array from a bottom reservoir (Fig. 1c). Our unprecedentedly large synthetic tree is easily primed by coiling within a pot of boiling water, in contrast to previous tree systems which required pumps^18^ or pressure bombs^3,25^. Further, we develop a model that solves for the negative Laplace pressure required to maintain hydration at any given flow rate. By showing that engineered trees (like natural trees) are capable of using nano-scale surface tension to power macro-scale hydraulic systems, this should enable the high-throughput filtration and elevation of water. We expect our tall and scalable synthetic tree will be useful for underground water extraction or for pumped storage hydropower, particularly when solar energy or waste heat are used to increase the evaporation-driven flow rate of the system.

## Results

### Scalable short tree

**Figure 2:**
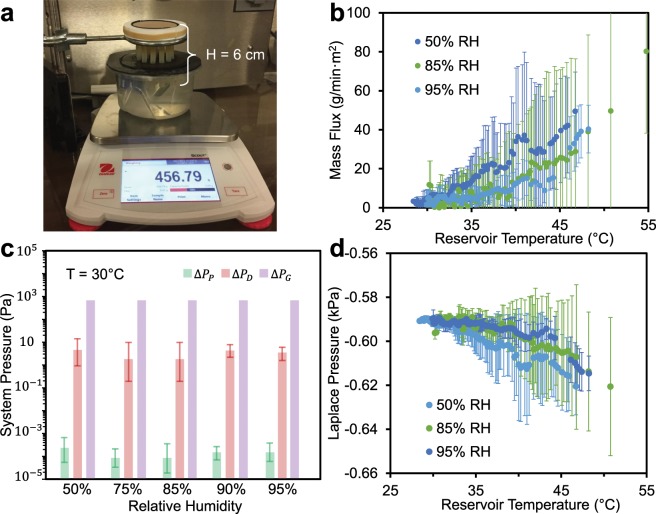
Demonstration of transpiration in a short (6 cm), scalable tree. (**a**) Photograph of the experimental setup, where 19 parallel tubes pump water from a bottom reservoir to an upper synthetic leaf. The transpiration rate is tuned by changing the ambient humidity using an environmental chamber and measured using a mass balance. (**b**) Transpiration mass flux of synthetic short trees as a function of the water temperature and ambient humidity. The pre-boiled water in the reservoir is cooling over the course of the experiment, such that time progresses from right-to-left along the x-axis and each data set represents a 3 h period. (**c**) Estimated pressure drops across the synthetic tree, where Δ*P*_P_ represents viscous losses along the tube array, Δ*P*_D_ is the pressure drop across the wetted leaf, and Δ*P*_G_ is the hydrostatic pressure column in each tube. Values of Δ*P*_P_ and Δ*P*_D_ were calculated to correspond to the flow rate measured at a reservoir temperature of 30 °C. (**d**) Estimated Laplace pressure as a function of the water temperature and ambient humidity, expressed as the sum of the three pressure drops found in (c). All values in (b–d) correspond to an average of three trials with error bars of one standard deviation.

First we demonstrate the viability of a synthetic tree that is scalable (i.e. multiple tube conduits), but short enough in height (*H* = 6 cm) to fit within an environmental chamber (Fig. 2a). Recall that previous vertical synthetic trees only employed a single micro-capillary; to attain scalable trees, it must be shown that the negative-pressure water can be transmitted across an array of conduits connected to a large-area nanoporous structure. Indeed, it has already been observed that when a large synthetic leaf is connected to only a single conduit, that dryout rapidly occurs within the leaf^36^.

A nanoporous ceramic disk, 54 mm in diameter and 7 mm thick, was chosen as the synthetic leaf material owing to its desirable properties of being commercially available, machinable, and large enough to embed an array of tubes. The ceramic disk exhibited a tortuous network of 80 nm-radius pores (Fig. S1). To embed tubes inside of the disk, cylindrical cavities were machined into its bottom face to allow for the insertion of one end of each tube. A water-proof adhesive was used to seal these connections air-tight. Tubes were 3.175 mm in inner diameter, to demonstrate the feasibility of using high-conductance tubing as synthetic xylem conduits (rather than microcapillaries). The height *H* of the tree is defined as the length of each vertical tube, including the portion embedded inside the nanoporous disk. Once the tubes were firmly embedded in the disk, the tree was boiled for several hours to charge with degassed water. After boiling, the disk was removed from the water and exposed to subsaturated air, while the bottom end of the tubes remained submerged within a sealed water reservoir (see Methods for more information).

When only one tube was embedded within the nanoporous disk, the system was prone to partial dry-out and low throughput due to an order of magnitude mismatch in the wetted diameters (Fig. S2a,b). While this partial dryout could not be directly observed, it was deduced from a continuous decrease in the transpiration rate over time. In contrast, when the same disk was outfitted with 7 or 19 tubes, the transpiration rate now stabilized to a constant value governed by the evaporation rate of water from the disk.

The 19-tube tree was tested at different relative humidities: 50, 75, 85, 90, or 95% RH, respectively (Fig. S2c,d). Figure 2(b) focuses on the 50, 85, and 95% cases for visual clarity. Throughout the paper, all transpiration rates are normalized by a generalized square meter of leaf area. Given that the exposed top area of our synthetic leaf is only 22.9 cm^2^, it is important to note that the mass flow rate (g/min) is smaller than the flux (g/min·m^2^) by a factor of 455. The transpiration rate did not vary appreciably for different ambient humidities, especially when taking the large trial-to-trial uncertainty into account. This relative insensitivity of the transpiration rate to the choice of ambient humidity was already analyzed in a recent report^36^. It is due to the tradeoff of lower humidities increasing the extent of menisci recession within the nanopores before they self-stabilize, which can choke the evaporative flow within the dried-out nanopores. This choking effect locally humidifies the air directly above the menisci, which cancels out the benefit of the drier ambient air with regards to the overall evaporation rate. Regardless, the mass flow measurements obtained here clearly show that transpiration can pump water against gravity, even up an array of large tubes and for a wide variety of ambient humidities.

### Modeling and physics

The negative water pressure generated in the leaf is prescribed by the curvature of the menisci^3^:

1 $${P}_{{\rm{L}}}=-\,\frac{2\gamma \,\cos \,\theta }{{r}_{{\rm{pore}}}},$$

where *γ* is the surface tension of water, *θ* is the contact angle of each meniscus with respect to the vertical side walls of the nanopores, and *r*_pore_ is the average radius of the pores in the leaf. The maximum possible Laplace pressure occurs when *θ* ≈ 0°, which for our case of *r*_pore_ ≈ 80 m corresponds to *P*_L,max_ ≈ −1.82 MPa.

The Kelvin equation quantifies the effective pressure acting on the menisci due to the mismatch in water activity between the subsaturated air and saturated water^25,37^:

2 $${P}_{{\rm{K}}}=\frac{RT}{{\nu }_{{\rm{liq}}}}\,\mathrm{ln}({a}_{{\rm{vap}}}),$$

where *R* is the universal gas constant, *T* is the temperature of the free interface, ${\nu }_{{\rm{liq}}}$ is the molar volume of liquid water, and ${a}_{{\rm{vap}}}={P}_{{\rm{vap}}}/{P}_{{\rm{sat}}}$ is the local activity of the water vapor immediately above the menisci. When *a*_vap_ < 1, a positive Kelvin pressure is exerted against the menisci. For the menisci to remain in physical equilibrium, an equivalently negative Laplace pressure must be generated within the liquid water^25^. However, physical equilibrium is impossible beneath a critical humidity where ${P}_{{\rm{K}}} > |{P}_{{\rm{L}},{\rm{\max }}}|$^25,33,34^. For the five humidities ranging from 50–95% RH tested here, the theoretical Kelvin pressures acting on the menisci range from 104 MPa to 8 MPa. In all cases, this exceeds the maximal Laplace pressure of $|{P}_{{\rm{L}},{\rm{\max }}}|\approx 1.82\,{\rm{MPa}}$ possible with our particular nanopores. This may lead one to expect system dryout, however, recent reports have shown that transpiration can be self-stabilizing when the menisci recede far enough within the nanopores that the local humidity is increased to a value where ${P}_{{\rm{K}}}\le |{P}_{{\rm{L}},{\rm{\max }}}|$^34,36^.

This necessary balance between the Kelvin and Laplace pressures to stabilize the free interface is well understood and has been thoroughly characterized^25,32–34^. Curiously lacking in the synthetic tree literature, however, is a second pressure balance required for conservation of mass. The Laplace pressure also represents the total pressure differential between the leaf and the atmospheric reservoir; for mass to be conserved, this pressure differential must correspond to the single value that produces a liquid mass flow rate equivalent to the evaporative flow rate $(\dot{m})$. In other words, we hypothesize here that $\dot{m}$ prescribes *P*_L_, which in turn will prescribe the local humidity required to stabilize the menisci $({P}_{{\rm{K}}}\approx |{P}_{{\rm{L}}}|)$.

There are three sources of pressure drops across our synthetic tree: viscous pressure drops across the nanoporous disk and the tubes, as well as hydrostatic pressure in the tubes. The pressure drop across the wetted disk can be estimated from Darcy’s law^34,38^:

3 $$\Delta {P}_{{\rm{D}}}=\frac{Qt}{\kappa A},$$

where *Q* is the volumetric flow rate, *t* = 7 mm and *A* = 22.9 cm^2^ are the thickness and cross-sectional area of the disk, and $\kappa =(\varphi {r}_{{\rm{pore}}}^{2})/(8\,\mu \tau )$ is the intrinsic permeability of the nanopores, where *φ* ≈ 0.32 is the porosity, *τ* ≈ 3.5 is the estimated tortuosity^39,40^, and *μ* is the viscosity of liquid water, which yield a hydraulic permeability of *κ* ≈ 8.2 × 10^−14^ m^2^/Pa · s. The viscous pressure drop across the tubes can be found using the Poiseuille equation^25,41^:

4 $$\Delta {P}_{{\rm{P}}}=\frac{8QH\mu }{\pi N{R}^{4}},$$

where *H* = 6 cm is the height of the tubes between the reservoir and the leaf, *R* = 1.59 mm is the radius of the tubes, and N is the number of the tubes in parallel. Finally, the hydrostatic pressure drop is $\Delta {P}_{{\rm{G}}}=\rho gH$. For mass to be conserved, $\Delta P=\Delta {P}_{{\rm{D}}}+\Delta {P}_{{\rm{P}}}+\Delta {P}_{{\rm{G}}}=|{P}_{{\rm{L}}}|$.

The pressure drops across our short synthetic tree were calculated for five relative humidities corresponding to the range of experimental conditions (Fig. 2c). The hydrostatic pressure term is two orders of magnitude larger than the Darcy pressure drop, which in turn is four orders of magnitude larger than the viscous pressure drop across the tubes. It makes sense that the viscous losses were dominant in the synthetic leaf, due to its nanoscale pores extending across a thick 7 mm disk. Regardless, the combined viscous losses were negligible compared to the hydrostatic pressure, even for the short tree. In Fig. 2(d), the Laplace pressure is calculated from the sum of all three pressure drops. The value of |*P*_L_| increased weakly with increasing temperature, due to the enhanced evaporation rate requiring a higher pressure drop across the tree to conserve mass. Quantitatively, the estimated suction pressure of *P*_L_ ≈ −0.6 kPa is far beneath the maximum possible value of *P*_L,max_ ≈ −1.82 MPa. This is due to the slow, diffusive evaporation rate of water from the leaf, which limits the flow rate (and by extension required pressure drop) across the tree. As a result of the low value of |*P*_L_| required to maintain hydration, the equivalently low Kelvin stress results in a local humidity of nearly 100% directly above the meniscus (Fig. S3). We will more fully explore this concept of evaporation-limited flow in a later section.

### Scalable tall tree

**Figure 3:**
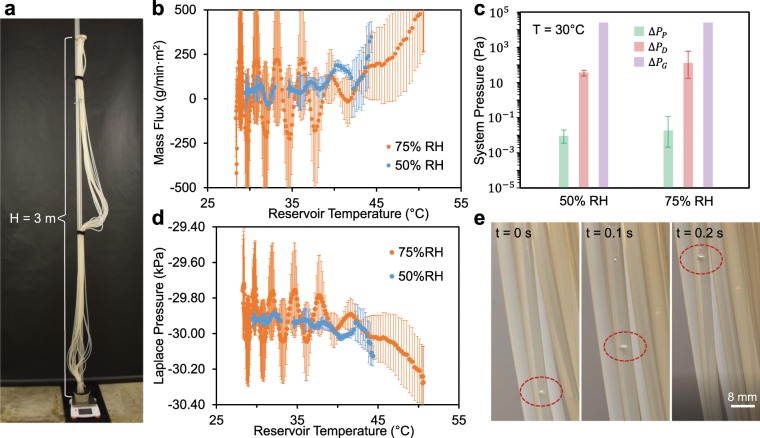
Demonstration of transpiration in a tall (3 m), scalable tree. (**a**) Photograph of the experimental setup, where the tubing and synthetic leaf are fixed to a tall vertical post. Transpiration is measured by placing the bottom reservoir on a mass balance, while the flow rate is controlled by tuning the ambient humidity of the tree growth chamber enclosing the entire tree. (**b**) Transpiration mass flux of the tall tree at 75% or 50% ambient humidities, as a function of the reservoir temperature recorded over 3 h experimental trials. (**c**) Pressure drops associated with the tall tree, calculated to match the flow rate measured at a reservoir temperature of 30 °C. (**d**) Calculated Laplace pressures of the tall tree at 75% and 50% ambient relative humidities, found as the sum of the three pressure drops. (**e**) During some trials, air bubbles (red circles) were periodically trapped within the tubes, resulting in the oscillatory backflow of the water. Graphical values in (**b**–**d**) are an average of three trials, while the error bars represent one standard deviation.

Now that transpiration has been demonstrated with a scalable synthetic tree, we attempt to increase the tree’s height by an order of magnitude. The tall tree setup uses the same nanoporous ceramic leaf connected to 19 tubes, but now these tubes exhibit a height of 3 m instead of 6 cm (Fig. 3a). The flexible tubing was coiled, such that the entire tall tree device could be submerged in a large pot (57 L volume) of boiling water heated by an industrial hot plate (see Methods). Once charged with degassed water, the ceramic leaf was elevated 3 m above the floor by climbing a ladder and fixing it to the top of a vertical post. To prevent the tubes from detaching from the elevated leaf due to their weight, they were Velcroed to the vertical post. The free end of the tubes remained submerged as they were transferred from the large pot to a small reservoir placed on a mass balance. This tall tree could not fit within our environmental chamber, so it was instead erected within a tree growth chamber where the humidity could be controlled.

For ambient humidities of 50% or 75% RH, the tall tree successfully exhibited transpiration over 3 h of operation. The 3 m tall water columns were always maintained within the tubes, while the mass balance measurements confirmed the flow of water up the tree to maintain leaf hydration (Fig. 3b). The mass flow rates were about twice as large for the tall tree compared to the short tree. We attribute this to the growth chamber’s use of convective air flows to maintain the room’s humidity, which will increase the evaporation rate from the leaf. As with the short tree, the mass flow rate was used to calculate the three pressure drops across the tree (Fig. 3c). The hydrostatic pressure was about an order of magnitude larger compared to the small tree, owing to the increase in tube height. The viscous losses across the tubes were about two orders of magnitude larger than before, due to both the increase in height and the enhanced flow rate. These changes do not alter the overall picture, where the hydrostatic term effectively dominates over both of the viscous terms. The sum of the pressure drops was an order of magnitude larger for the tall tree, resulting in a calculated Laplace pressure of about *P*_L_ ≈ −30 kPa (Fig. 3d).

For all three 50% RH trials and for one of the 75% RH trials, air bubbles were observed within some of the tubes (Fig. 3e). This was most likely due to an imperfect charging of the long tubes with the boiled water. The presence of these bubbles caused an oscillatory behavior, where the flow periodically switched between the typical upward flow and a reverse backflow into the reservoir. We attribute this periodic backflow to moments where bubbles completely block at least one tube. This would result in the water column beneath the bubble pressurizing to a positive gauge pressure: *ρgh*, where *h* is the height of the bubble with respect to the reservoir. With this positive pressure water cut off from the water above the bubble (which retains its negative gauge pressure), it has no choice but to flow back down toward the atmospheric reservoir. We hypothesize that the backflow always terminates eventually because the bubble cannot retain its perfect blockage. At least two factors could explain why a blockage cannot persist: the positive pressure of the underlying water could dislodge the bubble from the tube walls, or the bubble’s shape could change due to its expansion during backflow. We emphasize that these backflow events were quite minor: typically anywhere from ~1–10 g of water returned into the reservoir before reverting back to an upward transpiration (Fig. S4). Indeed, no obvious collapse of any of the liquid columns was observed, as there are 458 g of total water contained within the 19 tubes. It is remarkable that the liquid columns were, overall, able to be held in tension even during this intermittent backflow behavior.

For the 50% RH case, oscillatory flow occurred for all three trials, but this is difficult to see in Fig. 3b,d due to the destructive interference of averaging the trials together (as the oscillations are offset from each other). When separating the mass flow curves of the tall tree for each individual trial (Fig. S4), the oscillations can be clearly seen for each trial at 50% RH and for one of the three trials at 75% RH. Even for these cases where periodic backflow occurred, the tree still exhibited a net upward flow when looking at time scales exceeding the period of oscillation. It is in this sense, then, that we say that transpiration was always stable (despite the momentary disruptions caused by the bubbles). For the other two trials at 75% RH, which can also be seen separately in Fig. S4, no bubbles were observed and as a result there was no backflow at all. It is intriguing that the characteristic period of oscillation, 1/*f* ≈ 20 min, is similar to the previously reported oscillatory cycle of boiling in synthetic trees^31^. However, this is likely a coincidence, as the absolute pressure of the water in our tall tree is about ≈70 kPa (Fig. 3d), whereas the vapor phase is thermodynamically stable beneath ≈5–10 kPa for the water temperatures used here. Therefore, we do not expect any boiling behavior in our tall tree, although boiling could be envisaged when subjecting the leaf to higher evaporation rates (which would require a more negative value of *P*_L_).

### Regime maps

For all of the experiments performed here, the transpiration rate was limited by the diffusive evaporation rate from the leaf. This is because the pressure drop across the tree required to maintain hydration was far beneath the maximal possible Laplace pressure, such that only a negative gauge pressure was required (as opposed to a negative absolute pressure). We will call this regime evaporation-limited transpiration. We can envision a second regime, which we call flow-limited transpiration, where the flow rate is now constrained by the total pressure drop across the tree exceeding the maximal possible Laplace pressure. This flow-limited regime would occur at a sufficiently high flow rate, for example by increasing the evaporation rate from the leaves using radiation or resistive heating. This would result in increasingly large viscous pressure drops across the xylem and nanopores. Another way to switch from the evaporation-limited regime to the flow-limited regime is to change the geometry of the synthetic tree. For example, by making the tubes much taller, this would increase the hydrostatic pressure drop and viscous pressure drop across the tubes.

The critical inflection point between evaporation-limited versus flow-limited transpiration can be found by solving for the critical volumetric flow rate, *Q*_*c*_, where $\Delta {P}_{{\rm{D}}}+\Delta {P}_{{\rm{P}}}+\Delta {P}_{{\rm{G}}}=|{P}_{{\rm{L}},{\rm{\max }}}|$. Plugging in the expressions for each term:

5 $$\frac{{Q}_{{\rm{c}}}t}{\kappa A}+\frac{8{Q}_{{\rm{c}}}H\mu }{\pi N{R}^{4}}+\rho gH=\frac{2\gamma }{{r}_{{\rm{pore}}}},$$

where we assume our model tree system has vertical pores of tortuosity *τ* = 1 and a porosity of *φ* = 0.5. Our model system also assumes room temperature conditions, such that *μ* = 8.9 × 10^−4^, *ρ* = 1000 kg/m^3^, and *γ* = 0.0728 N/m. By solving Eq. 5 for ${\dot{m}}_{c}=\rho {Q}_{{\rm{c}}}$ over a wide range of tree geometries, regime maps can be used to predict the flow behavior of any tree.

**Figure 4:**
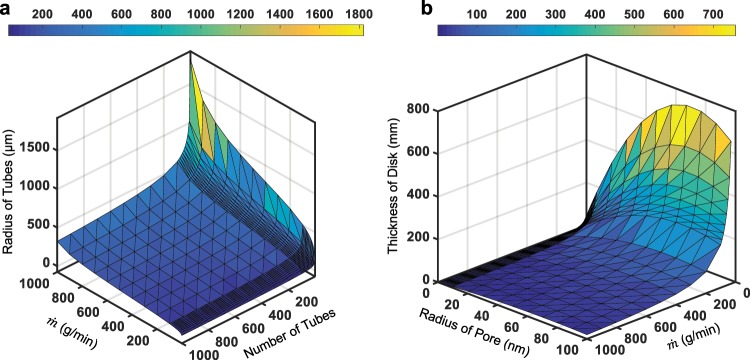
Regime maps demarcating an evaporation-limited regime, where the evaporation rate drives the transpiration rate, versus a flow-limited regime, where the flow up the tree cannot match the evaporation rate even when driven at the maximal Laplace pressure. The model tree system shown here assumed a fixed height of 100 m and leaf area of 1 m^2^. (**a**) The tube dimensions were varied for a fixed leaf thickness (1 cm), pore radius (20 nm), and leaf permeability (*κ* ≈ 2.8 × 10^−14^ m^2^/Pa·s). The phase space above the curve represents the evaporation-limited regime, while the flow-limited regime lies beneath. The color band is to help visualize the quantitative values of the tube radius (*μ*m) within the three-dimensional space. (**b**) Now the leaf properties were tuned, for a fixed tube radius (1 mm) and quantity of parallel tubes (1,000). The flow-limited regime is above the curve, with the evaporation-limited regime below. The color band now represents the values of the disk thickness (mm).

In Fig. 4a, the regime map shows the critical tube radius and number of tubes required to switch between the evaporation-limited and flow-limited behaviors for any given transpiration rate. For example, for an evaporation rate of $\dot{m}=1\,{\rm{k}}{\rm{g}}/min$, the critical tube radius is about 324 *μ*m when there are 1,000 tubes connected to a 1 m^2^ synthetic leaf where *r*_pore_ = 20 nm and *t* = 1 cm. Evaporation-limited flow will occur above this critical tube radius, whereas the tree is flow-limited beneath. Conversely, in Fig. 4b, the tube design is now fixed and the critical values are shown for the thickness and pore radius of the synthetic leaf material. Repeating the example of $\dot{m}=1\,{\rm{kg}}/{\rm{\min }}$, evaporation-limited flow occurs beneath a critical disk thickness of 0.61 mm for *r*_pore_ = 1 nm, for the fixed case of 1,000 tubes of size *R* = 1 mm attached to the 1 m^2^ synthetic leaf. In Fig. S5a–c, regime maps are shown for different tree heights (10 m, 50 m, and 100 m) across a phase space where $\dot{m}$, *N*, and *R* are varied. In Fig. S5d–f, regime maps for three different leaf areas (0.1 m^2^, 1 m^2^ and 10 m^2^) are provided where $\dot{m}$, *t* and *r*_pore_ are varied.

## Discussion

A brief discussion is warranted regarding the differences between natural leaves and our synthetic leaf. In a natural leaf, water vapor is confined within stomatal chambers and must diffuse into the ambient via dynamically controlled stomatal apertures^42–44^. As we showed in our recent report^36^, transpiration in a synthetic nanoporous leaf is stable even without any overlying stomata. Therefore, our present synthetic leaves lack any stomata for simplicity. Another key distinction is that, for natural leaves, an intermediate osmotic system exists between the water-saturated walls of the leaf cells and the xylem capillaries. In particular, the cellular wall matrix is generally infiltrated by a gel-like substance^45,46^. In contrast, the pure water within our minimal synthetic tree system is continuous, without any conflating osmotic processes. Finally, it is important to note that our synthetic tree is purely concerned with replicating the transpiration of water, and is not concerned with achieving photosynthesis or transporting sugars down the phloem.

In conclusion, we have demonstrated the stable transpiration of water up a tall synthetic tree that can scale to any area by virtue of using multiple tube conduits. This was achieved by embedding the upper ends of vertical tubes within a large-area nanoporous disk, while submerging the bottom ends in a water reservoir. Unlike previous synthetic trees, where the flows were confined to single micro-capillaries or substrate-bound micro-channels, our tree powers flow up an array of large-diameter tubes. Our largest tree exhibited a height of 3 m, with water flowing in parallel up 19 tubes of millimetric diameter. The synthetic trees were easily charged with degassed water, without the need for pumps or pressure bombs, by coiling within a pot of boiling water. While this boiling approach would not be conducive to ultra-tall or underground synthetic trees, it is extremely accessible for laboratory research or class projects regarding the characterization of large and flexible synthetic trees.

Across a wide range of water temperatures and ambient humidities, the synthetic trees demonstrated stable transpiration (net upward flow against gravity). Interestingly, a minor and periodic backflow of water back into the reservoir did occur for some trials, presumably due to a bubble blockage in the tubes, but these backflow events always switched back to stable transpiration. A comprehensive model was developed to show that the transpiration rate in any given synthetic tree is either operating in an evaporation-limited regime, constrained by the heat input, or a flow-limited regime, constrained by the maximal Laplace pressure driving the flow. Due to the purely diffusive evaporation used here, our present tree system always exhibited evaporation-limited transpiration. This resulted in only a negative gauge pressure being required to maintain hydration; in the future, we envisage using a heat source to increase the evaporation rate and achieve absolute negative pressures. By showing that synthetic trees, like natural trees, can use nanoscale surface tension to power macroscale hydraulic systems, we hope to broaden the length scales of devices that exploit water being held in tension.

## Methods

### Materials

To mimic leaf tissue, porous ceramic disks with an average pore size of *r*_pore_ ≈ 80 nm were purchased (Soilmoisture Equipment Corp). The disks were 54 mm in diameter, 7 mm thick, and 32% porous by volume. To mimic the xylem conduits, high-pressure silicone tubing (inner diameter 3.175 mm, outer diameter 6.35 mm) was attached to each ceramic disk by drilling small cavities into the disk’s bottom face, inserting one end of the tubes, and sealing with a waterproof silicone adhesive. The synthetic short trees were 6 cm tall with 1, 7 or 19 tubes connected to the ceramic disks. The synthetic tall tree was 3 m tall with 19 tubes, using the same ceramic disk as the short trees.

### Short tree setup

A pot of water was vigorously boiled for at least 3 h, prior to submerging a synthetic short tree for at least an hour. After the tree was fully charged with degassed water, the hot plate was turned off to stop the boiling. The tree was removed after letting the water cool for roughly one hour, with the exception of the free end of the xylem tubes, which remained submerged in a secondary reservoir that was submerged within the pot prior to removal. The leaf was placed on a 3D printed neoprene guard cell that was supported by a clamp attached to a vertical post. The secondary reservoir (i.e. filled plastic container) was placed on top of a mass balance (Scout Series, Ohaus) interfaced with a computer. The reservoir was sealed with a rubber lid, such that water in the reservoir could not escape by evaporation but only by transpiration up the tube(s). This seal, while sufficient to halt any appreciable evaporation, was not air-tight to prevent a vacuum from forming as the enclosed water evaporated. As the degassed water was still warm from the boiling process, the temperature of water within the reservoir was measured by submerging a digital data logger in the reservoir underneath the leaf (HOBO Pendant). The synthetic tree was tested for 3 h at 50, 75, 85, 90, and 95% RH (three trials each), exposed to the room temperature environment and the ambient humidity of the surrounding air was set using a large humidity chamber (Electro-Tech Systems, Inc.) that enclosed the setup.

### Tall tree setup

Water was boiled for at least 5 h in a large 57 L aluminum pot, of diameter 45 cm and height 41 cm, placed on an industrial hot plate (61 cm × 91 cm, Valad Electric Heating Corp.). The large tree was then completely submerged, by coiling the flexible tubes, and boiled for at least 2 h. The synthetic leaf was fixed atop a 3 m tall vertical post, while the tubes were also bound to the post to prevent their weight from causing detachment from the leaf. The bottom reservoir was prepared and placed on a mass balance in an identical fashion to the short tree. The tall tree was tested for at least 3 h in a humidity-controlled tree growth chamber at Virginia Tech, at either 50% RH and 75% RH (three trials each humidity). The setup was placed on top of a mass balance (Scout Series, Ohaus) interfaced with a computer and a digital data logger in the reservoir underneath the leaf (HOBO Pendant) was used to measure the evolving temperature of the reservoir.

### SEM imaging

A ceramic disk was cut both horizontally and vertically to create cross-sections. A scanning electron microscope (LEO (Zeiss) Field Emission SEM) at Virginia Tech’s Nanoscale Characterization and Fabrication Laboratory was used to visualize the pore structure of the cross-sections.

## Supplementary information

Supplementary Information.

## References

[1] Koch GW, Sillett SC, Jennings GM, Davis SD (2004). The limits to tree height. Nature.

[2] Holbrook, N. M. & Zwieniecki, M. A. *Vascular Transport in Plants* (Academic Press, 2011).

[3] Stroock AD, Pagay VV, Zwieniecki MA, Holbrook NM (2014). The physicochemical hydrodynamics of vascular plants. Annu. Rev. Fluid Mech..

[4] Scholander PF (1968). How mangroves desalinate seawater. Physiol. Plant..

[5] Parida AK, Jha B (2010). Salt tolerance mechanisms in mangroves: a review. Trees.

[6] Kim K, Seo E, Chang SK, Park TJ, Lee SJ (2016). Novel water filtration of saline water in the outermost layer of mangrove roots. Sci. Rep..

[7] Paudel I, Naor A, Gal Y, Cohen S (2015). Simulating nectarine tree transpiration and dynamic water storage from responses of leaf conductance to light and sap flow to stem water potential and vapor pressure deficit. Tree Physiol.

[8] Novick KA, Miniat CF, Vose JM (2016). Drought limitations to leaf-level gas exchange: results from a model linking stomatal optimization and cohesion-tension theory. Plant Cell Environ..

[9] Schenk, H. J. *et al*. Xylem surfactants introduce a new element to the cohesion–tension theory. *Plant Physiol*. 1–41 (2016). 10.1104/pp.16.01039 PMC5291718 27927981

[10] Bentrup F-W (2017). Water ascent in trees and lianas: the cohesion-tension theory revisited in the wake of otto renner. Protoplasma.

[11] Lens F, Cochard ATH, Sperry JS, Jansen S, Herbette S (2013). Embolism resistance as a key mechanism to understand adaptive plant strategies. Curr. Opin. Plant Biol.

[12] Caupin F (2012). Exploring water and other liquids at negative pressure. J. Phys.: Condens. Matter.

[13] Brown HR (2013). The theory of the rise of sap in trees: some historical and conceptual remarks. Phys. Perspect..

[14] Cai J, Tyree MT (2014). Measuring vessel length in vascular plants: can we divine the truth? History, theory, methods, and contrasting models. Trees.

[15] Petit G, Crivellaro A (2014). Comparative axial widening of phloem and xylem conduits in small woody plants. Trees.

[16] Kim HK, Park J, Hwang I (2014). Investigating water transport through the xylem network in vascular plants. J. Exp. Bot..

[17] Dixon HH, Joly J (1895). On the ascent of sap. Philos. Trans. R. Soc. B.

[18] Thut H (1928). Demonstration of the lifting power of evaporation. Ohio J. Sci..

[19] Hayward ATJ (1970). Mechanical pump with a suction lift of 17 metres. Nature.

[20] Lee M, Lim H, Lee J (2017). Fabrication of artificial leaf to develop fluid pump driven by surface tension and evaporation. Sci. Rep..

[21] Lamb M, Koch GW, Morgan ER, Shafer MW (2015). A synthetic leaf: the biomimetic potential of graphene oxide. Proc. of SPIE.

[22] Juncker D (2002). Autonomous microfluidic capillary system. Anal. Chem..

[23] Borno RT, Steinmeyer JD, Maharbiz MM (2009). Charge-pumping in a synthetic leaf for harvesting energy from evaporation-driven flows. Appl. Phys. Lett..

[24] Li JM (2011). A bio-inspired micropump based on stomatal transpiration in plants. Lab Chip.

[25] Wheeler TD, Stroock AD (2008). The transpiration of water at negative pressures in a synthetic tree. Nature.

[26] Noblin X (2008). Optimal vein density in artificial and real leaves. Proc. Natl. Acad. Sci..

[27] Wheeler TD, Stroock AD (2009). Stability limit of liquid water in metstable equilibrium with subsaturated vapors. Langmuir.

[28] Vincent O, Marmottant P, Quinto-Su PA, Ohl CD (2012). Birth and growth of cavitation bubbles within water under tension confined in a simple synthetic tree. Phys. Rev. Lett..

[29] Duan C, Karnik R, Lu MC, Majumdar A (2012). Evaporation-induced cavitation in nanofluidic channels. Proc. Natl. Acad. Sci. U.S.A.

[30] Li J (2012). A microfluidic pump/valve inspired by xylem embolism and transpiration in plants. PLOS ONE.

[31] Vincent O, Sessoms DA, Huber EJ, Guioth J, Stroock AD (2014). Drying by cavitation and poroelastic relaxations in porous media with macroscopic pores connected by nanoscale throats. Phys. Rev. Lett..

[32] Pagay V (2014). A microtensiometer capable of measuring water potentials below −10 MPa. Lab Chip.

[33] Chen IT (2016). Stability limit of water by metastable vapor-liquid equilibrium with nanoporous silicon membranes. J. Phys. Chem. B.

[34] Vincent O, Szenicer A, Stroock AD (2016). Capillarity-driven flows at the continuum limit. Soft Matter.

[35] Comtet J, Jensen KH, Turgeon R, Stroock AD, Hosoi AE (2017). Passive phloem loading and long-distance transport in a synthetic tree–on–a–chip. Nature Plants.

[36] Shi W (2019). Self-stabilizing transpiration in synthetic leaves. ACS Appl. Mater. Interfaces.

[37] Skinner LM, Sambles J (1972). The Kelvin equation–a review. J. Aerosol Sci..

[38] Whitaker S (1986). Flow in porous media I: A theoretical derivation of Darcy's law. Transport Porous Med..

[39] Gruener S, Wallacher D, Greulich S, Busch M, Huber P (2016). Hydraulic transport across hydrophilic and hydrophobic nanopores: Flow experiments with water and n-hexane. Phys. Rev. E.

[40] Crossley PA, Schwartz LM, Banavar JR (1991). Image-based models of porous media: Application to vycor glass and carbonate rocks. Appl. Phys. Lett..

[41] Giordano R, Salleo A, Salleo S, Wanderlingh F (1978). Flow in xylem vessels and Poiseuille’s law. Botany.

[42] Pittermann J (2010). The evolution of water transport in plants: an integrated approach. Geobiology.

[43] I. R. Cowan. *Stomatal Behaviour and Environment*, vol. **4** (Academic Press, 1978).

[44] Gao Q, Zhao P, Zeng X, Cai X, Shen W (2002). A model of stomatal conductance to quantify the relationship between leaf transpiration, microclimate and soil water stress. Plant Cell Environ..

[45] Pickard WF (1981). The ascent of sap in plants. Prog. Biophys. Molec. Biol..

[46] Rand RH (1983). Fluid mechanics of green plants. Annu. Rev. Fluid Mech..

