# Author Correction: Passive water ascent in a tall, scalable synthetic tree

**DOI:** 10.1038/s41598-021-81423-0

**Published:** 2021-01-26

**Authors:** Weiwei Shi, Richard M. Dalrymple, Collin J. McKenny, David S. Morrow, Ziad T. Rashed, Daniel A. Surinach, Jonathan B. Boreyko

**Affiliations:** 1 Department of Biomedical Engineering and Mechanics, Virginia Tech, Blacksburg, Virginia 24061 USA; 2 Department of Mechanical Engineering, Virginia Tech, Blacksburg, Virginia 24061 USA

Correction to: *Scientific Reports* 10.1038/s41598-019-57109-z, published online 14 January 2020

This Article contains errors.

After publication of this Article, the Authors discovered an error in the code used to calculate the Darcy pressure drop (ΔP_D_) across the nanoporous synthetic leaf. While Eq. 3 in the Article is correct, this code issue resulted in numerical values that were approximately four orders of magnitude too small. Additionally, the value of t used for Eq. 3 is 3.5 mm, not 7 mm as stated in the Article, to account for the fact that the vertical tubes were embedded halfway inside of the nanoporous disk.

The Authors recalculated all values of ΔP_D_, which led to change of the Darcy pressure drop which is now approximately one order of magnitude larger than the hydrostatic pressure drop for the short tree and roughly the same order of magnitude for the tall tree. Given that the negative Laplace pressure, P_L_, is equivalent to the sum of all three pressure drops, these values were also updated. Now the Laplace pressure is of order P_L_ ~ − 10 kPa for the short tree and ranges from ~ − 10 kPa to ~ − 100 kPa for tall tree. Note that in the absence of the oscillatory flows caused by an air bubble, P_L_ ≈ − 50 kPa is typical for the tall tree. This indicates that boiling is still not expected to be thermodynamically favorable, as the absolute pressure of water in the tree is usually greater than 10 kPa. Finally, considering that the Kelvin pressure is balanced with the Laplace pressure, this correction also updates the local humidity directly above the menisci. However, due to the slow (diffusive) evaporation rate, the resulting local humidity is still very close to 100%, so this change is not appreciable.

As a result of these changes, data shown in parts of Figures 2 and 3 and in Supplementary Figure 3 is corrected. The correct Figures 2, 3 and S3 appear below as Figures 1, 2 and 3 respectively.

In addition, in the Results section under subheading ‘Modeling and physics.’,

“The maximum possible Laplace pressure occurs when *θ* ≈ 0°, which for our case of *r*pore ≈ 80 m corresponds to *P*L,max ≈ − 1.82 MPa.”

should read:

“The maximum possible Laplace pressure occurs when *θ* ≈ 0°, which for our case of *r*pore ≈ 80 nm corresponds to *P*L,max ≈ − 1.82 MPa.”

“where *Q* is the volumetric flow rate, *t* = 7 mm and *A* = 22.9 cm^2^ are the thickness and cross-sectional area of the disk,”

should read:

“where *Q* is the volumetric flow rate, *t* = 3.5 mm and *A* = 22.9 cm^2^ are the thickness and cross-sectional area of the disk,”

“The hydrostatic pressure term is two orders of magnitude larger than the Darcy pressure drop, which in turn is four orders of magnitude larger than the viscous pressure drop across the tubes. It makes sense that the viscous losses were dominant in the synthetic leaf, due to its nanoscale pores extending across a thick 7 mm disk. Regardless, the combined viscous losses were negligible compared to the hydrostatic pressure, even for the short tree.”

should read:

“The Darcy pressure drop is about one order of magnitude larger than the hydrostatic pressure drop for the short tree.”

“The value of |*P*L| increased weakly with increasing temperature, due to the enhanced evaporation rate requiring a higher pressure drop across the tree to conserve mass. Quantitatively, the estimated suction pressure of *P*L ≈ − 0.6 kPa is far beneath the maximum possible value of *P*L,max ≈ − 1.82 MPa.”

should read:

“The value of |*P*L| increased with increasing temperature, due to the enhanced evaporation rate requiring a higher pressure drop across the tree to conserve mass. Quantitatively, the estimated suction pressure of *P*L ≈ − 10 kPa is far beneath the maximum possible value of *P*L,max ≈ − 1.82 MPa.”

Furthermore, under subheading ‘Scalable tall tree.’,

“The viscous losses across the tubes were about two orders of magnitude larger than before, due to both the increase in height and the enhanced flow rate. These changes do not alter the overall picture, where the hydrostatic term effectively dominates over both of the viscous terms.”

should read:

“The Darcy pressure drop is roughly the same order of magnitude for the tall tree.”

“The sum of the pressure drops was an order of magnitude larger for the tall tree, resulting in a calculated Laplace pressure of about *P*L ≈ − 30 kPa (Fig. 3d).”

should read:

“The sum of the pressure drops was an order of magnitude larger for the tall tree, resulting in a calculated Laplace pressure of about *P*L ≈ − 10 kPa to ∼ − 100 kPa (Fig. 3d).”

“However, this is likely a coincidence, as the absolute pressure of the water in our tall tree is about ≈70 kPa (Fig. 3d), whereas the vapor phase is thermodynamically stable beneath ≈5–10 kPa for the water temperatures used here.”

should read:

“However, this is likely a coincidence, as the absolute pressure of the water in our tall tree is about greater than 10 kPa (Fig. 3d), whereas the vapor phase is thermodynamically stable beneath ≈5–10 kPa for the water temperatures used here.”

Finally, under subheading ‘Regime maps.’,

“Our model system also assumes room temperature conditions, such that *μ* = 8.9 × 10^−4^, *ρ* = 1000 kg/m^3^, and *γ* = 0.0728 N/m.”

should read:

“Our model system also assumes room temperature conditions, such that *μ* = 8.9 × 10^(− 4) Pa·s, *ρ* = 1000 kg/m^3^, and *γ* = 0.0728 N/m.”

These changes do not affect the overall conclusions of the Article.

**Figure 1:**
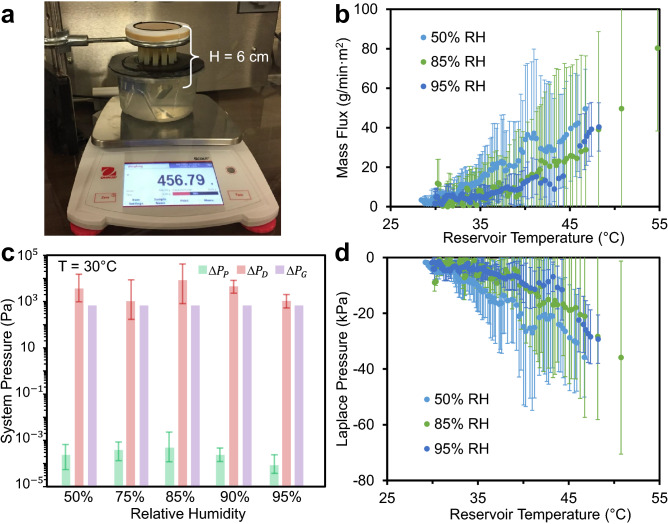
Demonstration of transpiration in a short (6 cm), scalable tree. (**a**) Photograph of the experimental setup, where 19 parallel tubes pump water from a bottom reservoir to an upper synthetic leaf. The transpiration rate is tuned by changing the ambient humidity using an environmental chamber and measured using a mass balance. (**b**) Transpiration mass flux of synthetic short trees as a function of the water temperature and ambient humidity. The pre-boiled water in the reservoir is cooling over the course of the experiment, such that time progresses from right-to-left along the x-axis and each data set represents a 3 h period. (**c**) Estimated pressure drops across the synthetic tree, where Δ*P*_P_ represents viscous losses along the tube array, Δ*P*_D_ is the pressure drop across the wetted leaf, and Δ*P*_G_ is the hydrostatic pressure column in each tube. Values of Δ*P*_P_ and Δ*P*_D_ were calculated to correspond to the flow rate measured at a reservoir temperature of 30 °C. (**d**) Estimated Laplace pressure as a function of the water temperature and ambient humidity, expressed as the sum of the three pressure drops found in (c). All values in (b–d) correspond to an average of three trials with error bars of one standard deviation.

**Figure 2:**
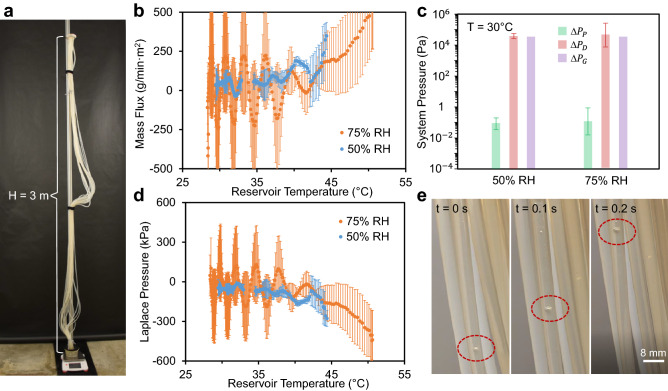
Demonstration of transpiration in a tall (3 m), scalable tree. (**a**) Photograph of the experimental setup, where the tubing and synthetic leaf are fixed to a tall vertical post. Transpiration is measured by placing the bottom reservoir on a mass balance, while the flow rate is controlled by tuning the ambient humidity of the tree growth chamber enclosing the entire tree. (**b**) Transpiration mass flux of the tall tree at 75% or 50% ambient humidities, as a function of the reservoir temperature recorded over 3 h experimental trials. (**c**) Pressure drops associated with the tall tree, calculated to match the flow rate measured at a reservoir temperature of 30 °C. (**d**) Calculated Laplace pressures of the tall tree at 75% and 50% ambient relative humidities, found as the sum of the three pressure drops. (**e**) During some trials, air bubbles (red circles) were periodically trapped within the tubes, resulting in the oscillatory backflow of the water. Graphical values in (**b**–**d**) are an average of three trials, while the error bars represent one standard deviation.

**Figure 3:**
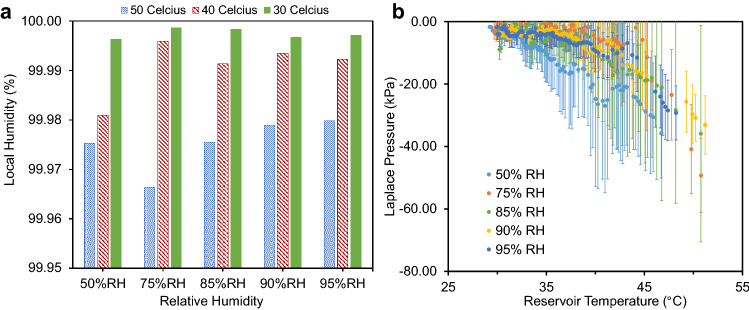
Local humidities and Laplace pressures for the short tree experiments. **(a)** The local humidity immediately above the water menisci within the nanopores approached 100%. This is due to the slow, diffusive transpiration rate, which requires a very low Laplace pressure to conserve mass. By balancing this small Laplace pressure with the corresponding Kelvin pressure, a high local humidity is obtained. Physically, this high humidity is achieved by the menisci partially retreating to concentrate vapor within the nanopores, at which point the menisci position stabilizes. **(b)** Estimated Laplace pressures generated by the concave water menisci under five ambient relative humidities. Values were obtained by summing the three pressure drops across the tree required to achieve a theoretical ow rate equal to the measured transpiration rate.

