# Supplementary material for: Passive water ascent in a tall, scalable synthetic tree
Source: Sci Rep. 2020 Jan 14;10:230. doi: 10.1038/s41598-019-57109-z (PMC6959229; doi:10.1038/s41598-019-57109-z)
Supplement: Supplementary file 1 — Supplementary Information. [file 41598_2019_57109_MOESM1_ESM.pdf]

Supplementary Information: Passive water ascent in a tall, scalable synthetic tree

Weiwei Shi[1], Richard M. Dalrymple[1], Collin J. McKenny[1], David S. Morrow[1], Ziad T. Rashed[1], Daniel A. Surinach[1], and Jonathan B. Boreyko[1, 2]

[1]Department of Biomedical Engineering and Mechanics

[2]Department of Mechanical Engineering, Virginia Tech, Blacksburg, Virginia 24061, United States

1 Nanoporous synthetic leaf

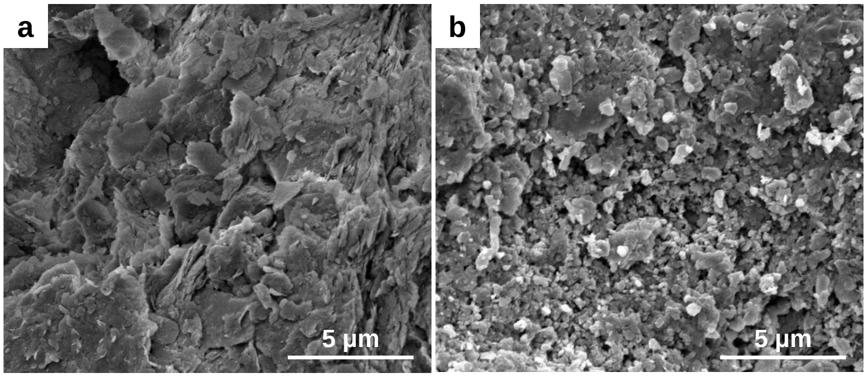

Supplementary Figure S1: SEM images of the nanoporous ceramic disk (synthetic leaf). (a) Pore morphology of a cross-section, as obtained by cutting across the face of the disk. (b) Pore morphology of a second cross-section, by cutting through the middle of the rim of the disk. The disks were 54 mm in diameter, 7 mm thick, and the porosity was 32% by volume with an average pore radius of 80 nm (as specified by the manufacturer).

2 Scalable short tree

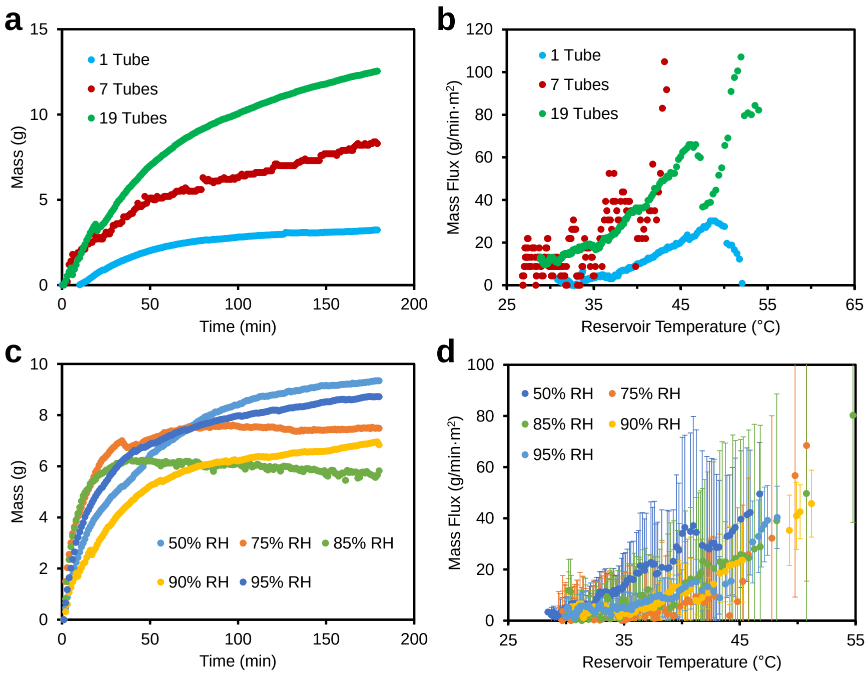

Supplementary Figure S2: Transpiration mass flux of the short tree for all five relative humidities. (a) Mass flow across the short tree with 1, 7 or 19 tubes. (b) Mass flux of the short tree with 1, 7 or 19 tubes, this time normalized by the reservoir temperature. Aside from noise in the data, there is no obvious difference in the transpiration rate when comparing the 7-tube and 19-tube cases, indicating the evaporation-limited regime. Time progresses from right-to-left along the x-axis as the pre-boiled water is cooling down. (c) Mass flow of the short tree with 19 tubes under different ambient relative humidities. (d) Mass flux of the 19-tube short tree, normalized by water temperature.

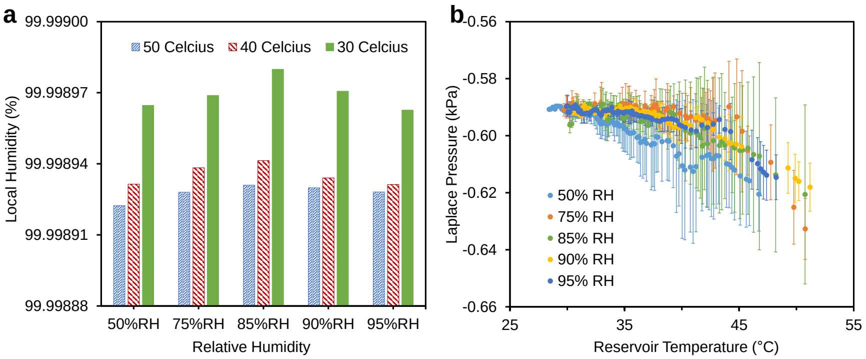

Supplementary Figure S3: Local humidities and Laplace pressures for the short tree experiments. (a) The local humidity immediately above the water menisci within the nanopores approached 100%. This is due to the slow, diffusive transpiration rate, which requires a very low Laplace pressure to conserve mass. By balancing this small Laplace pressure with the corresponding Kelvin pressure, a high local humidity is obtained. Physically, this high humidity is achieved by the menisci partially retreating to concentrate vapor within the nanopores, at which point the menisci position stabilizes. (b) Estimated Laplace pressures generated by the concave water menisci under five ambient relative humidities. Values were obtained by summing the three pressure drops across the tree required to achieve a theoretical flow rate equal to the measured transpiration rate.

3 Scalable tall tree

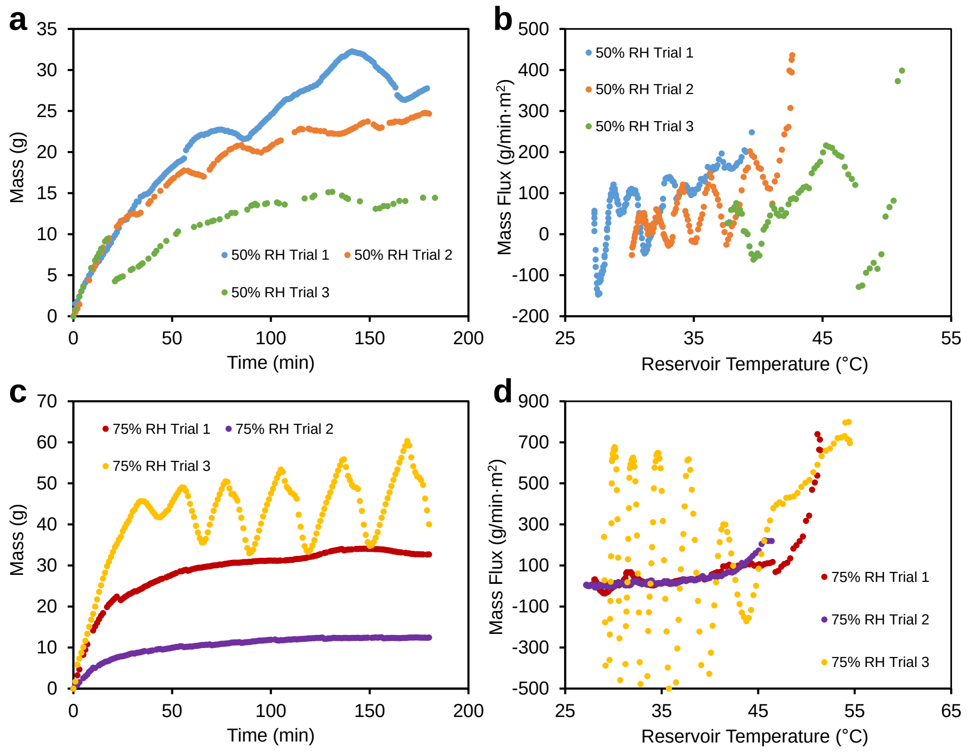

Supplementary Figure S4: Raw data for transpiration rates in the tall synthetic tree, where each individual trial is shown. (a) Mass flow up a 19-tube tall tree at 50% humidity. (b) Mass flux normalized by water temperature, again for the 50% humidity. Oscillations indicate periodic backflow induced by entrapped air bubbles, as described in the manuscript. (c) Mass flow up a 19-tube tall tree at 75% humidity. (d) Mass flux as a function of water temperature for the 75% humidity case, where the oscillatory flow was only observed for one of the three trials.

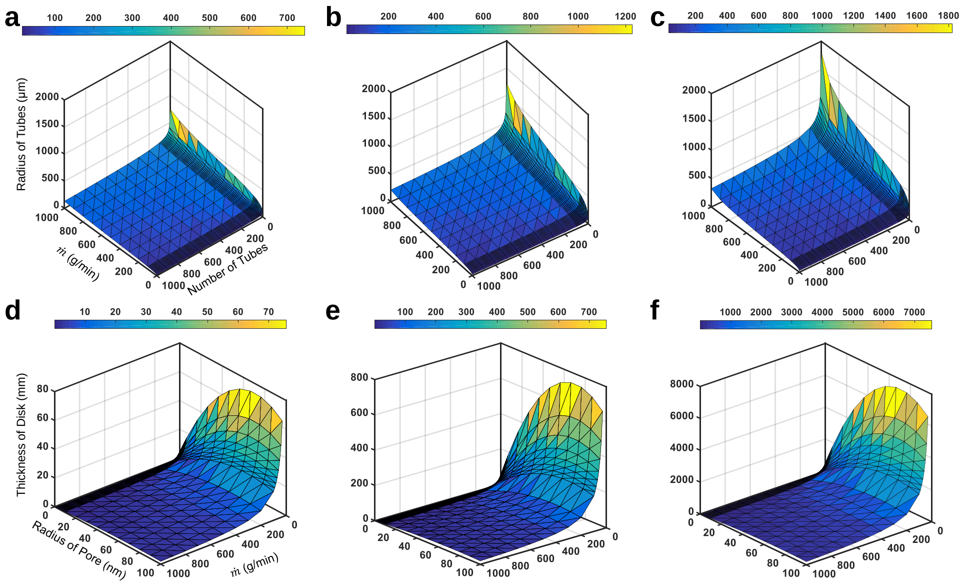

Supplementary Figure S5: Regime maps demarcating an evaporation-limited regime, where the evaporation rate drives the transpiration rate, versus a flow-limited regime, where the flow up the tree cannot match the evaporation rate even when driven at the maximal Laplace pressure. (a–c) Design maps for the xylem, where the radius and number of tubes are varied along with the transpiration rate. The leaf is fixed at a thickness of 1 cm, porosity of 50%, and pore radius of 20 nm, while the tree height is 10 m, 50 m or 100 m, respectively. The regions above the curves represent the evaporation-limited regime, while the regions beneath the curves are the flow-limited regime. Color bands represent the magnitude of the tube radius (μm). (d–f) Design map for the synthetic leaf, where the pore radius and leaf thickness are varied along with the transpiration rate. The xylem tubes are fixed at a radius of 1 mm and a quantity of 1,000 in parallel, while the cross-sectional areas of the leaves are 0.1 m^2, 1 m^2 and 10 m^2, respectively. The evaporation-limited regime now lies beneath the curves, while the flow-limited regime is above. Each color band now represents the thickness of the synthetic disk (mm).
